# Role of Venous Sampling in the Diagnosis of Endocrine Disorders

**DOI:** 10.3390/jcm7050114

**Published:** 2018-05-14

**Authors:** Ryan W. England, Eliza B. Geer, Amy R. Deipolyi

**Affiliations:** 1 Interventional Radiology Service, Memorial Sloan Kettering Cancer Center, New York, NY 10065, USA; ryan.w.england@gmail.com; 2 Multidisciplinary Pituitary and Skull Base Tumor Center, Memorial Sloan Kettering Cancer Center, New York, NY 10065, USA; geere@mskcc.org; 3 Endocrine Service, Memorial Sloan Kettering Cancer Center, New York, NY 10065, USA

**Keywords:** venous sampling, primary aldosteronism, Cushing’s disease, hyperparathyroidism, inferior petrosal sinus, adrenal vein

## Abstract

Venous sampling is the gold standard for localizing abnormal hormone secretion in several endocrine disorders. The most common indication for venous sampling is in the workup of primary aldosteronism, adrenocorticotropic hormone-dependent Cushing’s syndrome, and hyperparathyroidism. In experienced hands, venous sampling is safe and accurate. This review discusses the role of venous sampling in the workup of endocrine disease, describing the underlying anatomy and pathophysiology, as an understanding of these concepts is essential for technical and clinical success.

## 1. Introduction

Venous sampling is a powerful diagnostic tool that serves as the gold standard for localizing several endocrine disorders while assisting in the medical and surgical management for these patients. As these procedures are used more frequently throughout care centers around the world, collaboration between interventional radiology, endocrinology, and surgical teams is essential.

A number of endocrine disorders require venous sampling for localization. More than 25% of Americans are affected by hypertension [1], of which 15% is due to primary aldosteronism (PA). Therefore, an estimated 12 million people in the US alone have hypertension caused by PA. Primary hyperparathyroidism (PHPT) is the third most common endocrine disorder, influencing 3 to 4 per 1000 patients [2]. Cushing’s disease (CD), which is Cushing’s syndrome due to a pituitary ACTH-secreting tumor, is the most common cause (67%) of endogenous CS, and has a reported incidence of about 2–3 per million per year. There is also an emerging role for venous sampling in pancreatic, ovarian, and prostate disease.

To assist in the workup of these diseases, interventional radiologists perform venous sampling to localize sites of abnormal hormonal secretion, using minimally invasive catheters directed to the venous drainage of specific glands for sampling and hormone analysis. Adrenal venous sampling (AVS) has become the gold standard test for localizing excess aldosterone in PA, assisting in both medical and surgical planning for these patients. Understanding laterality is essential for managing PA, with bilateral disease indicating medical management, while unilateral disease prompts surgical intervention. Another venous sampling technique, bilateral inferior petrosal sinus sampling (BIPSS), is also a gold standard diagnostic assay, used for localizing CS in the workup of adrenocorticotropic hormone (ACTH)-dependent disease. While noninvasive imaging and laboratory testing often lack sensitivity and can confound diagnosis, BIPSS is both sensitive and specific, able to differentiate between pituitary and ectopic ACTH secretion [3]. Roughly 5–10% of patients with PHPT who have undergone surgery return with persistent or recurrent postoperative hypercalcemia [4]. For these patients, parathyroid venous sampling (PVS) is the most sensitive test for localizing the ectopic or missed adenoma.

## 2. Pathophysiology

The basic principle behind many endocrinopathies is a disruption of an endocrine axis that leads to an excess or insufficiency of one or more hormones. Understanding the underlying pathophysiology is essential when performing venous sampling.

### 2.1. Cushing’s Disease

Endogenous CS is a disorder in the hypothalamus-pituitary-adrenal axis that leads to excess cortisol production. With normal hypothalamus-pituitary-adrenal axis function, the hypothalamus releases corticotropin-releasing hormone (CRH), which then acts on the anterior pituitary. ACTH is then released by the pituitary into the bloodstream, causing the adrenal glands to secrete cortisol, which then inhibits hypothalamic and pituitary secretion via a negative feedback mechanism. Two-thirds of cases of abnormal, endogenous cortisol secretion are due to CD, wherein a pituitary corticotroph tumor secrets ACTH without hormonal stimulation and despite elevated cortisol levels. Unrestricted ACTH secretion causes excess circulating cortisol levels and subsequent CS. One-third of endogenous CS cases are due to either adrenal adenomas/carcinomas or ectopic ACTH-secreting tumors, which result in similar sequelae. As a result of excess cortisol, patients with CS demonstrate typical signs and symptoms including central obesity, moon facies, buffalo hump, proximal muscle weakness, alopecia, hirsutism, hypertension, hyperglycemia, amenorrhea, and impotence [5].

Anatomically, the pituitary gland is drained by hypophyseal veins that empty into a plexiform venous network, which in turn flow laterally into intercavernous and cavernous sinuses. These then drain into the superior petrosal sinus and inferior petrosal sinus (IPS), which empties into the internal jugular vein (IJV). The diameter of the IPS is about 2–4 mm within the jugular foramen, making it the most proximal vein to the pituitary gland that can safely hold a microcatheter for ACTH measurements while minimizing dilution from other veins downstream [6]. IPS sampling is performed by cannulating each IPS and comparing ACTH concentrations from the IPSs to peripheral samples drawn from the inferior vena cava.

### 2.2. Primary Aldosteronism

PA involves dysregulation of the renin-angiotensin-aldosterone system (RAAS), which plays an important role in blood pressure regulation. The RAAS involves secretion of renin by renal juxtaglomerular cells in reaction to decreased blood flow. Renin converts angiotensinogen in the liver to angiotensin I, which is converted to angiotensin II by angiotensin converting enzyme (ACE) found in lung. Angiotensin II stimulates the adrenal cortex to secrete aldosterone. Normally, increased angiotensin II and aldosterone levels act as negative feedback, suppressing further renin secretion through systemic vasoconstriction and extracellular volume expansion [7,8].

Most cases (98%) of PA are due to aldosterone-producing adenomas (35–40%) or bilateral adrenal hyperplasia (60%) [9]. Excess aldosterone acts on the distal tubules of the kidney to increase salt and water reabsorption and potassium excretion, resulting in volume expansion with hypertension as well as hypokalemic metabolic acidosis. As a result, patients with PA often have hypokalemia and hypertension resistant to standard antihypertensive medications, as well as nonspecific symptoms including muscle weakness, cramping, palpitations, and polyuria with polydipsia [10].

As aldosterone is secreted by the adrenal glands, AVS measures this hormone level bilaterally by cannulating the left and right adrenal veins (AVs). While the left AV usually drains into the left renal vein through a common trunk shared with the inferior phrenic vein, the right AV usually drains directly into the posterolateral inferior vena cava superior to the right renal vein. AVS can determine whether PA is due to a unilateral aldosterone-producing adenoma, which is surgically managed, or a bilateral adrenal hyperplasia, which is managed with medical treatment.

### 2.3. Primary Hyperparathyroidism

The parathyroid glands involve a feedback system that regulates circulating calcium. In contrast to other endocrinopathies that are regulated by trophic hormones, parathyroid glands are regulated by free, ionized calcium. In the setting of low calcium, the parathyroids release parathyroid hormone (PTH) that acts on bone (for calcium resorption), kidneys (to increase calcium reabsorption and activate 1,25-dihydroxyvitamin D), and the intestines (to increase calcium absorption). The resulting increase in calcium acts as negative feedback on the parathyroid glands to decrease PTH secretion [11]. Conditions causing hyperparathyroidism can be primary through an independent, unprompted overproduction of PTH, or secondary (and less commonly tertiary) in patients typically with chronic renal insufficiency. PHPT is usually caused by a single parathyroid adenoma (85%) or less commonly involving multiple glands (15%, either primary hyperplasia or multiple adenoma). Rarely (1–2%) PHPT is caused by parathyroid carcinoma [12,13]. Hereditary forms of PHPT are rare, with the most common cases arising from the multiple endocrine neoplasia (MEN) type 1 and 2 syndromes or the familial hyperparathyroidism-jaw tumor (HPT-JT) syndrome [14].

Most patients with PHPT are asymptomatic, with clinically silent hypercalcemia being discovered incidentally during blood tests. However, in some patients, traditional symptoms of PHPT may follow the classic phrase of “renal stones, painful bones, abdominal groans, and psychic moans.” Prolonged hypercalcemia can affect the renal system (causing polyuria, polydipsia, and nephrolithiasis), musculoskeletal system (producing osteoporosis, fractures, and weakness), gastrointestinal system (as seen by peptic ulcers, pancreatitis, and gallstones), and the central nervous system (causing depression, lethargy, and seizures) [15,16].

PTH typically drains through parathyroid veins via the thyroid plexus, but the venous anatomy, as well as the location of parathyroid glands, can vary [17]. In most patients, three pairs of thyroid veins merge to form a valve-less thyroid plexus, which in turn drains into the internal jugular (IJ) as well as the brachiocephalic veins. Because glands may reside in the mediastinum, it is important to note the location of the thymic and internal mammary veins (IMV).

## 3. Noninvasive Workup

Prior to any venous sampling procedure, proper noninvasive workup is performed to help with diagnosis and to optimize procedure planning.

### 3.1. Cushing’s Disease

**Figure 1:**
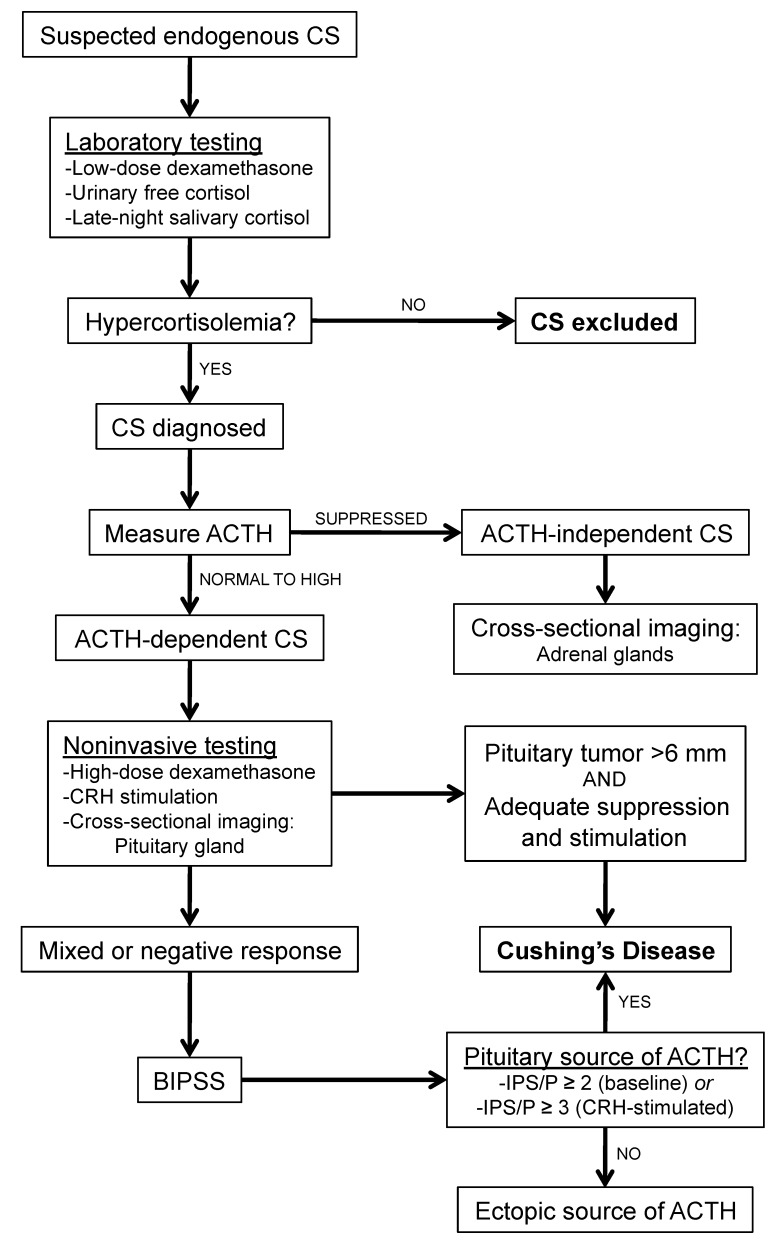
Workup of Cushing’s syndrome. Suspected endogenous Cushing’s syndrome is evaluated in a stepwise manner. BIPSS is indicated when noninvasive testing is inconclusive for diagnosing Cushing’s disease which involves a pituitary source of ACTH. CS: Cushing’s syndrome; ACTH: adrenocorticotropic hormone; CRH: corticotropin-releasing hormone; IPS: inferior petrosal sinus; P: peripheral; BIPSS: bilateral inferior petrosal sinus sampling.

In patients with suspected endogenous CS, workup is performed in a stepwise manner (Figure 1). Hypercortisolemia is first evaluated with laboratory tests including low-dose dexamethasone suppression test, 24-h urinary free cortisol, and late-night salivary cortisol measurements [18]. If hypercortisolemia is confirmed and endogenous CS is diagnosed, plasma ACTH is measured. Low plasma ACTH concentrations suggest ACTH-independent CS. In such patients, adrenal imaging is the next step to localize the source of excess adrenal cortisol production. Alternatively, patients with high or normal plasma ACTH levels have ACTH-dependent CS, which may be due to a pituitary or ectopic source.

If ACTH-dependent CS is diagnosed, a high-dose dexamethasone test or CRH stimulation test can be performed to help localize the source of excess ACTH production. Serum cortisol levels that do not suppress appropriately to high-dose dexamethasone is suggestive of an ectopic source (such as carcinoid and neuroendocrine tumors, pheochromocytoma, gastrinoma, medullary thyroid, pancreatic carcinoma, or bronchioloalveolar carcinoma) [19]. However, this test has been found to have a sensitivity of 60% and specificity of 80% [20]. Similarly, while the CRH stimulation test induces most pituitary corticotroph tumors to increase ACTH secretion and cortisol levels as a result, many ectopic ACTH-secreting tumors also respond to CRH [21].

Finally, pituitary imaging such as MRI is also unreliable in diagnosing CD, as roughly 40% of CD patients have tumors that are too small for detection by MRI [22], and there is a high prevalence (10–20%) of nonfunctioning pituitary “incidentalomas” [23,24]. As a result, only in cases where imaging shows a large (≥6 mm) lesion with supportive laboratory and clinical findings can be considered diagnostic of CD. Otherwise, patients should undergo BIPSS with CRH stimulation for further evaluation.

### 3.2. Primary Aldosteronism

**Figure 2:**
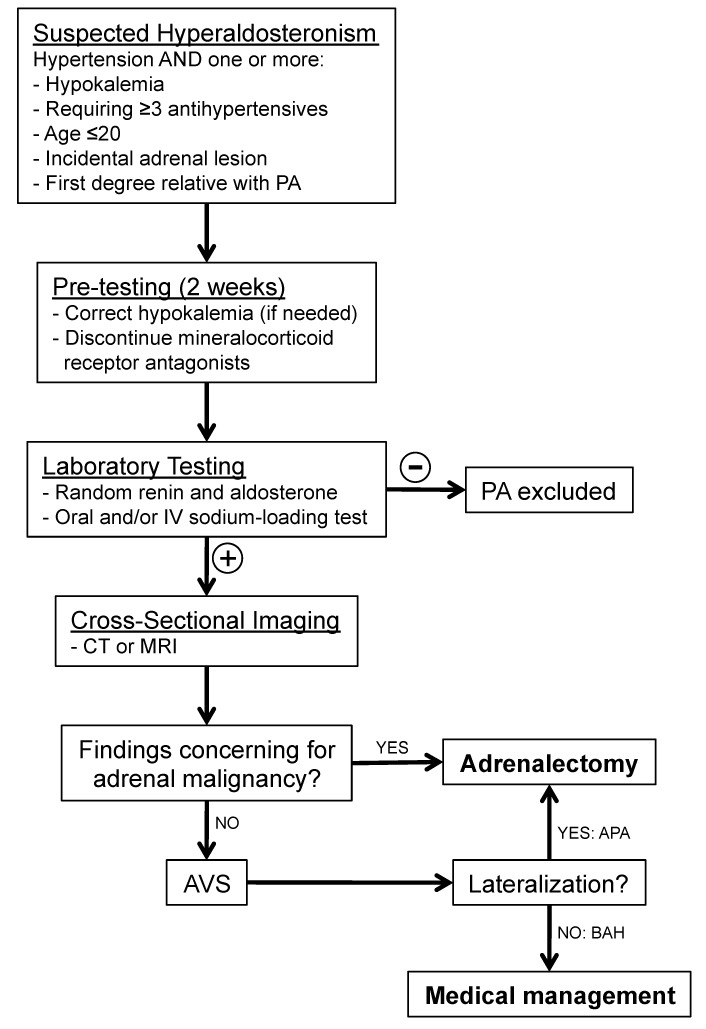
Workup of hyperaldosteronism. Primary aldosteronism is evaluated in a stepwise manner. If imaging fails to demonstrate suspected adrenal malignancy, AVS is performed to diagnose a unilateral source of excess aldosterone secretion, prior to adrenalectomy. PA: primary aldosteronism; AVS: adrenal vein sampling; APA: aldosterone-producing adenoma; BAH: bilateral adrenal hyperplasia.

A comprehensive diagnostic workup is indicated for all patients suspected to have PA (Figure 2). This includes all hypertensive patients who also present with either hypokalemia, resistance to standard medication regimens, young age (≤20 years), an incidental adrenal lesion found on cross-sectional imaging, or a first-degree relative with PA [25]. For these patients, hypokalemia is first corrected, and mineralocorticoid receptor antagonists such as spironolactone are discontinued for at least 2 weeks. Initial screening consists of random morning plasma aldosterone and renin levels. Elevated aldosterone/renin ratios are highly specific for PA (exact threshold values laboratory specific). Other confirmatory tests include an oral sodium-loading test, an intravenous (IV) saline infusion test, a fludrocortisones suppression test, and a captopril challenge [26].

Following laboratory-confirmed PA, cross-sectional imaging (CT or MRI) is performed primarily for identifying large adrenal masses that may be concerning for malignancy, which would then forgo the need for AVS. Imaging such as CT and MRI should typically not be used to determine laterality, and has been shown in numerous studies to have low sensitivity and specificity, with results that would either cause inappropriate adrenalectomy or inappropriate exclusion from adrenalectomy in over a third of patients [27]. ^131^I-6β-iodomethyl-19-norcholesterol adrenal scintigraphy has been suggested to be useful in lateralizing aldosterone-secreting adenomas, though with an accuracy of only 77% has not gained popularity in the workup of PA [28]. Therefore, for patients with PA who do not have findings concerning for adrenal malignancy, AVS is indicated to identify patients with unilateral versus bilateral disease. Certain patients may not benefit from AVS, such as those with normokalemia and normal CT or MR imaging of the adrenal glands, as there is only a 6% prevalence of unilateral hyperaldosteronism in this group [29]. Patients younger than 35 years with hypokalemia, marked hyperaldosteronemia, and unilateral adrenal lesions with imaging consistent with a cortical adenoma on CT scan may also not need AVS before proceeding to adrenalectomy [25,29].

### 3.3. Primary Hyperparathyroidism

**Figure 3:**
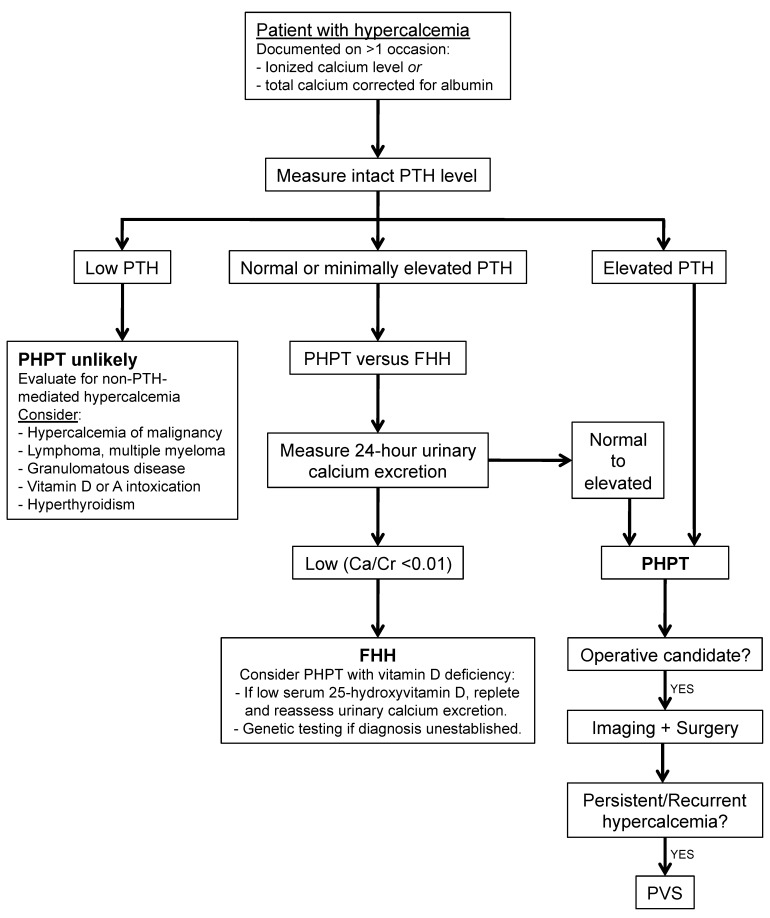
Workup of hypercalcemia. Primary hyperparathyroidism is diagnosed in patients with elevated parathyroid hormone and normal to elevated 24-h urinary calcium excretion. PTH: parathyroid hormone; PHPT: primary hyperparathyroidism; FHH: familial hypocalciuric hypercalcemia; PVS: parathyroid venous sampling.

For patients with suspected PHPT, diagnosis is based on clinical and biochemical studies (Figure 3). Hypercalcemia, either corrected using total serum calcium and albumin level or ionized calcium level, is typically documented on more than one occasion before further workup. Next, intact PTH is measured, with elevated levels diagnostic of PHPT. Minimally elevated or upper-normal levels of PTH should prompt a 24-h urinary calcium excretion to distinguish between PHPT versus autosomal dominant familial hypocalciuric hypercalcemia, with possible 25-hydroxyvitamin D levels and/or genetic testing if the resulting calcium/creatinine clearance ratio is indeterminate [30].

For patients who are operative candidates with confirmed PHPT, imaging is first used to locate one or more hyperfunctioning parathyroid glands. As minimally invasive parathyroid surgery is the approach of choice to allow for unilateral neck dissections with subsequent reduced operating times and complication rates, preliminary localization is crucial [31]. The first-line diagnostic tool is sestamibi scintigraphy (technetium-99-sestamibi scan) with sestamibi single photon emission computed tomography (SPECT), which has a sensitivity of 70–81% and a PPV of 91–95%. Other options depending on available radiologic expertise include ultrasound (sensitivity 64–91%, PPV 83–96%), 4D-CT (sensitivity 83–95%, PPV 88–99%), and 11c-methionine positron emission topography and computed tomography (MET-PET-CT; sensitivity 79–90%, PPV 93–94%) [32,33].

Despite good imaging and surgical techniques, approximately 5–10% of patients who undergo surgery for PHPT will have persistent or recurrent hypercalcemia due to either missed, additional, or ectopic adenomas [34]. These patients may benefit from PVS, especially when noninvasive imaging techniques are unconvincing or fail to localize. PVS has been shown to be able to identify hyperfunctioning parathyroid tissue even when other imaging modalities are negative [35].

## 4. Procedure and Results Interpretation

### 4.1. Bilateral Inferior Petrosal Sinus Sampling for Cushing’s Disease

For patients without a definitive diagnosis of CD from imaging and laboratory testing, BIPSS with CRH stimulation is indicated. This is accomplished by sampling ACTH before and after CRH administration, from the inferior vena cava or femoral vein (peripheral specimen) and from both IPSs. Patients are given conscious sedation to allow for continuous monitoring of possible symptoms that might indicate a procedural complication. Two sheaths are advanced into both right and left common femoral veins, a smaller 5-French on the left and a larger 6-French on the right to allow for venous sampling at the common femoral during the procedure. IV heparin (usually 3000–5000 units) is then administered to prevent cavernous sinus or other deep vein thromboses.

**Figure 4:**
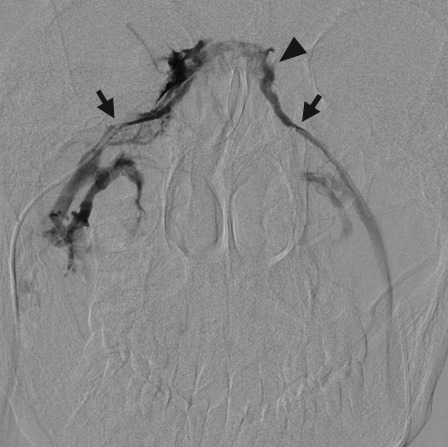
Inferior petrosal sinus venography. During bilateral inferior petrosal sinus sampling, the inferior petrosal sinuses are cannulated with microcatheters (arrows). Gentle hand injection demonstrates contralateral reflux (arrowhead) when the IPS is adequately cannulated.

Two 5-French catheters are then advanced through the femoral vein sheaths and into contralateral IJ veins. Afterward, 2.8-French microcatheters are advanced through the guiding catheter, directed medially at the level of C1–C2 to access the orifice of the IPS, and positioned symmetrically. Optimal catheter placement is beyond the anterior condylar vein to prevent dilution. Once microcatheter positions are confirmed by showing reflux into the contralateral IPS with gentle hand injection (Figure 4), baseline ACTH measurements are taken from both IPSs as well as the right femoral vein via the sheath.

CRH is administered peripherally (given as a bolus of 100 μg, or at a dose of 1 μg/kg, slow IV push over 30 s), with repeat ACTH specimens collected at 3, 5, 10, and 15 min after injection. These samples should be collected in separate tubes and placed on ice before being transported to the laboratory. If CRH is not available, desmopressin, a vasopressin synthetic analog, can be used to stimulate pituitary hormone secretion. Given at a dose of 10 μg IV, a series of 18 patients showed similar laboratory findings compared to CRH stimulation, with a sensitivity of 95% [36]. While these findings are promising and may prove to become standard practice given desmopressin’s low cost compared to CRH, further studies are needed to confirm safety and efficacy. Finally, catheters and sheaths are removed, and manual compression is applied at entry sites to obtain homeostasis before transferring the patient to the recovery room where they should rest and be observed for 3–4 h.

Interpretation of results involves calculating the ratio of IPS to peripheral (P) ACTH levels assessed at each time point. CD is confirmed by an IPS/P ratio of ≥2:1 at baseline, and/or a CRH-stimulated IPS/P ratio of ≥3:1 [37] in the setting of hypercortisolemia, usually defined by an elevated 24-h urinary free cortisol collected on the day prior to testing. Overall, the technical success rate for bilateral cannulation is high and has been shown to be about 88% with 2/3 of technical failures resulting in unilateral cannulation that was still diagnostic of CD [38]. The sensitivity and specificity of BIPSS approach 100%, especially with CRH stimulation incorporated into the procedure [39]. To consider further reducing false negatives, prolactin levels can be used to demonstrate correct catheter positioning and confirm the adequacy of sampling [40].

In the hands of experienced interventional radiologists, BIPSS is extremely safe, with very low complication rates. Most commonly, groin hematomas may occur in less than 5% of patients, similar to other procedures that require femoral venous access. Rarely, thromboembolic events such as cavernous sinus thrombosis or deep vein thrombosis may occur, which can potentially lead to PE [41]. These events are minimized by routine heparinization for patients prior to advancing catheters. Very rarely (1 or 2 occasions reported over hundreds of sampling procedures), serious complications of brainstem hemorrhage or infarction have been reported [42].

### 4.2. Adrenal Vein Sampling for Primary Aldosteronism

For patients with laboratory-confirmed PA and imaging findings that do not suggest malignancy, AVS is performed to distinguish laterality prior to surgery. Mineralocorticoid receptor antagonists are discontinued for several (at least 2) weeks prior to AVS avoid confounding sampling measurements.

For venous sampling, the adrenal and peripheral (IVC or common femoral) veins can be sampled either sequentially or simultaneously. Simultaneous sampling holds an advantage of eliminating potential temporal changes in hormone secretion, but requires bilateral common femoral venous access and is technically more difficult to maintain stable access to both AVs at the same time. To avoid these challenges while maintaining diagnostic accuracy, most centers use sequential sampling with the addition of cosyntropin stimulation, which stabilizes hormonal variation [43].

For the sequential sampling approach, a single 5- or 6-French sheath is first positioned in the right common femoral vein. Intravenous heparin should be considered to prevent thrombosis, especially if pre-imaging studies suggest a prolonged procedure time due to difficult cannulations secondary to aberrant anatomy. Continuous cosyntropin infusion is started at least 30 min prior to any venous sampling. Suggested dosing involves adding 250 mcg cosyntropin to 500 mL isotonic saline, then infusing at 100 mL/h.

**Figure 5:**
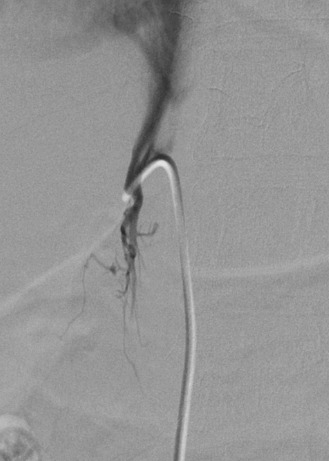
Right adrenal venography. During adrenal vein sampling, the right adrenal vein is cannulated with a 4- or 5-French catheter. Gentle hand injection demonstrates downward or laterally sloping veins that do not communicate with the hepatic veins.

**Figure 6:**
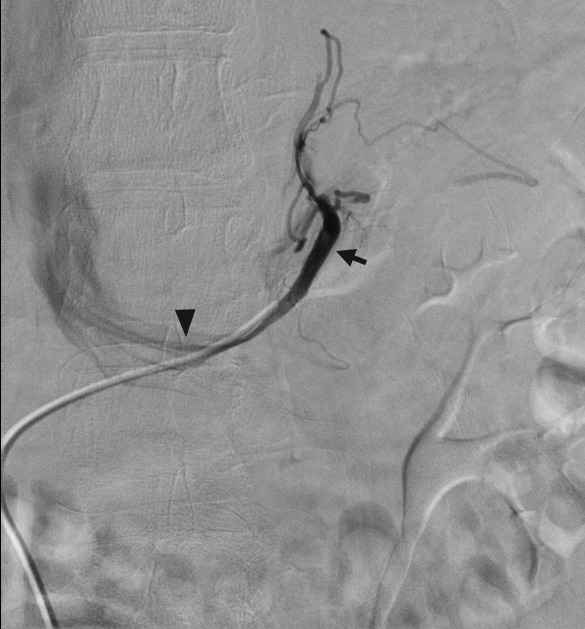
Left adrenal venography. The left adrenal vein (arrow) merges with the inferior phrenic vein. The common trunk empties into the left renal vein (arrowhead). In this venogram, the left adrenal is hypertrophied due to a hormone-secreting nodule.

Next, a 4- or 5-French catheter is passed through the sheath and directed to each AV. The right AV (Figure 5) originates from the IVC and is technically more difficult to cannulate, and can result in a non-diagnostic study due to failed cannulation [44]. An initial vena cavagram in the expected position of the right AV origin can be taken, with subsequent gentle hand injections to help confirm the catheter position. The left AV arises from the left renal vein, sharing a trunk with the inferior phrenic vein (Figure 6). Sampling can be performed on either the common trunk, or a microcatheter can be advanced to select the AV specifically. Finally, a peripheral specimen can be collected from either the common femoral vein (via the sheath) or the IVC. The samples are not put on ice, and are transported in individual tubes to the laboratory.

The technical success rate exceeds 90–95% depending on patient anatomy and operator experience [45,46]. To improve technical success, multiple specimens can be taken from any candidate right AV, along with one or two samples from the left AV and/or common venous trunk. Additionally, rapid cortisol testing may be utilized during the procedure immediately following right AV sampling to confirm AV cannulation. This testing usually takes around 30–60 min and can be performed during left AV cannulation [47].

To interpret the results of AVS, both cortisol and aldosterone are measured from venous samples, with cortisol serving as a positive control. As the adrenal gland will produce both during stimulation, a properly cannulated AV will show an adrenal vein to peripheral vein cortisol ratio of at least ≥3 for AVS with cosyntropin stimulation or ≥2 if performed without [47]. This ratio is usually many times greater when the AV is adequately cannulated, and many algorithms call for a cutoff of 5 under stimulation [43]. Next, aldosterone levels are normalized to corresponding cortisol levels (A_LAV_/C_LAV_ and A_RAV_/C_RAV_). Each side’s A/C ratio is then divided by the other to produce the lateralization index (LI). An LI of ≥4 is compatible with a unilateral source of aldosterone (aldosterone-secreting adenoma) while an LI closer to 1 suggests bilateral adrenal hyperplasia.

AVS is a safe procedure with complication rates less than 1%. The most common, as with each venous sampling procedure, is groin hematoma. Other rare complications such as adrenal vein and gland hemorrhage can result from strong AV injections, and present as pain that may persist for 1–2 days. Major complications such as adrenal insufficiency, hypertensive crisis, and thrombosis are also rare and inversely correlated to operator experience.

Recent studies have described “super-selective” adrenal venous sampling (ssAVS), whereby tributaries of each adrenal vein (superior, lateral, and inferior) are sampled with a microcatheter [48,49,50,51]. This technique may allow for detection of segmental adrenal lesions, allowing for partial bilateral adrenalectomies that spares normal adrenal tissue. Theoretically, ssAVS could help identify patients with bilateral focal lesions that are surgical candidates who otherwise would have been diagnosed with bilateral adrenal hyperplasia.

### 4.3. Parathyroid Hormone Venous Sampling for Primary Hyperparathyroidism

Patients with persistent or recurrent PHPT after surgery may require PVS to localize abnormal glands. Because most patients have had prior invasive neck exploration prior to PVS, understanding the surgical procedures and imaging findings is essential [52,53].

**Figure 7:**
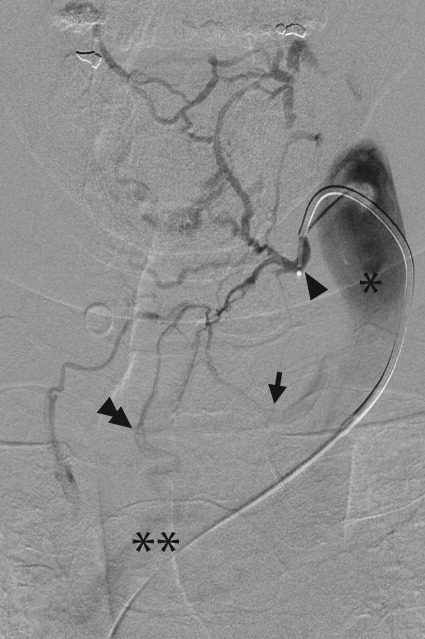
Thyroidal venography. During parathyroid hormone venous sampling, the thyroidal veins are key to target as most abnormal tissue will be located in the neck. The superior and medial thyroidal veins empty into the internal jugular vein (asterisk). In this image, the superior thyroidal vein (arrowhead) has been cannulated and hand injection with prolonged imaging demonstrates the orifices of the middle thyroidal vein (arrow) at the caudal internal jugular vein, and the inferior thyroid trunk (double arrowhead), which empties into the left brachiocephalic vein (double asterisk).

A 5- or 6-French venous sheath is placed in the right common femoral vein. A long sheath can be used to assist in catheter exchanges while also avoiding manipulations through the right atrium. A 4- or 5-French catheter is then advanced, with a peripheral venous sample from the SVC taken as a control. Prior to accessing the thyroid veins, samples can be taken from the azygous, thymic, and superior intercostal veins, as well as bilateral internal mammary, brachiocephalic, and vertebral veins. Selective catheterization of the superior, middle, and inferior thyroid veins is critical. As the thyroid plexus does not contain valves, a retrograde venogram through any thyroid vein may be taken to identify the anatomy prior to individual cannulation (Figure 7). Various catheter shapes along with microcatheters may be required for selective catheterization in PVS [54]. About 2–3 mL samples are taken from each site and sent to the laboratory. As there are several venous samples being taken, in addition to good labeling techniques, it is helpful to send samples with a venous diagram to ensure proper interpretation of the results and subsequent localization.

In the interpretation of the results for PVS, elevations in PTH for selected veins as compared to peripheral vein samples are helpful for regionalizing parathyroid glands. Ratios showing elevations of 1.5-, 2.5-, and 3-fold increases in PTH have a PPV of 72, 75, and 83%, respectively [55]. When real-time rapid PTH assays are available to enable super-selective venous sampling during PVS, a PPV of 93% and sensitivity of 86% has been shown for gradients ≥2 [35]. Overall, PVS has the highest sensitivity for localization of an ectopic or missed parathyroid adenoma following surgery compared to other imaging modalities, reaching over 85% in studies [56,57].

Complication rates from PVS are uncommon and similar to that of other venous procedures, such as groin hematoma, venous thrombosis, arrhythmias, contrast reaction, and renal failure [58].

## 5. Emerging Venous Sampling Procedures

Additional venous sampling techniques are being used to diagnose other diseases, including insulinoma, ovarian tumors, and prostate cancer.

After confirming a diagnosis of insulinoma, the most common cause of hypoglycemia from endogenous hyperinsulinism, conventional imaging techniques such as ultrasound and CT will fail to detect the tumor in up to 30% of patients [59]. With the addition of selective arterial calcium stimulation test (SACST), also known as arterial stimulation venous sampling (ASVS), this detection rate rises to over 94% [60,61].

This dynamic test utilizes the fact that calcium stimulates hyperfunctioning beta cells within insulinomas to release insulin while having little to no effect on normal beta cells. With ASVS, a venous catheter is placed at the right hepatic vein for sampling. A second catheter is then selectively placed at various arteries that feed the pancreas, including the gastroduodenal (GDA), superior mesenteric (SMA), and splenic arteries (SA), as well as the proper hepatic artery (PHA), with sequential calcium gluconate injections. Venous samples are taken before arterial calcium gluconate injection, and then every 30 s up to 3 min, with 5 min delays between each arterial stimulation to allow insulin levels to return to baseline. A two-fold or greater rise in insulin above baseline is considered diagnostic, with a rise following GDA, SMA, or SA stimulation suggestive of a antero-superior pancreatic head, postero-inferior pancreatic head, or pancreatic body/tail tumor, respectively, while an insulin rise following PHA injection is suggestive of hepatic metastases. ASVS, as a result, plays an important role in operative localization.

Similarly, selective venous catheterization may assist women with androgen-secreting ovarian tumors, when conventional modalities such as US, CT, and MR are unsuccessful in identifying ovarian lesions. Following catheter-directed bilateral ovarian vein testosterone sampling, localization of the tumor was found to be correctly categorized in 66% of cases [62].

The use of a prostate-specific antigen (PSA) in the detection of prostate cancer is being increasingly used, but with limited positive predictive value (PPV). For PSA levels over 10 ng/mL, PPV is 42–64%. However, for PSA levels between 4 and 10 ng/mL, PPV drops dramatically to approximately 25%, with 75% of cancers detected within these values being organ-confined and potentially curable [63]. To assist in diagnosing patients with prostate adenocarcinoma and borderline elevation of PSA, catheter-directed venous sampling of bilateral internal iliac veins has shown promise, with free to total PSA percentage (fPSA%) being significantly higher compared to peripheral venous sampling [64].

## 6. Conclusions

Catheter-directed venous sampling has become the gold standard for localizing several endocrine disorders. In testing for ACTH-dependent CS, primary aldosteronism, and primary hyperparathyroidism, venous sampling is a sensitive and safe modality. An understanding of the procedure, including the relative anatomy and subsequent results, is crucial for proper diagnosis and operative planning. Accuracy is high in experienced hands. Given the relative rarity of certain procedures, such as BIPSS, it is important that venous sampling be performed in centers of excellence with multidisciplinary teams specialized in endocrine disorders. Emerging procedures are currently being developed to aid in the diagnosis of other disorders where conventional imaging techniques are inconclusive. Their eventual role in diagnostic algorithms is yet to be determined.
